# Ozone Gas for Low Cost and Environmentally Friendly Desulfurization of Mute Grape Must

**DOI:** 10.3390/foods11101405

**Published:** 2022-05-12

**Authors:** Marina Delogu, Brunella Ceccantoni, Roberto Forniti, Isabella Taglieri, Chiara Sanmartin, Fabio Mencarelli, Andrea Bellincontro

**Affiliations:** 1 Department of Innovation for Biological, Agro-Food and Forest Systems, University of Tuscia, 01100 Viterbo, Italy; marina.delogu@gmail.com (M.D.); b.ceccantoni@unitus.it (B.C.); forniti@unitus.it (R.F.); bellin@unitus.it (A.B.); 2 Department of Agriculture, Food and Environment, University of Pisa, 56124 Pisa, Italy; chiara.sanmartin@unipi.it (C.S.); fabio.mencarelli@unipi.it (F.M.); 3 Interdepartmental Research Center “Nutraceuticals and Food for Health”, University of Pisa, Via del Borghetto 80, 56124 Pisa, Italy

**Keywords:** ozone, desulfurization, mute must, color, tannins, ascorbic acid

## Abstract

Ozone is widely used for storage and processing facilities and food sanitization. In this research, ozone was tested as an alternative to high temperature vacuum must desulfurization in order to make a more sustainable process. Bubbling ozone in highly sulfited red must (mute must) at two treatment temperatures, a significant reduction in total and free sulfites from around 1000 mg/L to 200 and 120 mg/L at 20 and 10 °C, respectively, was observed in 24 h, but already after 4 h the concentration was halved. Air flushing of the mute must did not reduce the SO_2_ content. To evaluate the potential ozone effect on polyphenol oxidation, we carried out the ozone treatment on a water solution with tannins, ascorbic acid, or potassium metabisulfite (MBK) as single and as mixture. In 1 h, 2/3 of sulfite disappeared with the treatment, but the reduction was greater with ascorbate and tannins; the same was observed for ascorbate, whereas tannins decreased to a lesser extent when combined with ascorbate and MBK. Taken together, the results indicate that ozone could be an environmentally friendly, low cost, treatment for desulfurization, especially for white must, and is also easy to use by small wineries.

## 1. Introduction

In the OIV (Organization Internationale de la Vigne et du Vin) International Code of Oenological Practice, the paragraph N.2.2 Preserved grape must (16/70 and 5/88) gives the following definition: ‘Fresh grape must whose alcoholic fermentation has been prevented by one of the following oenological procedures: sulfiting or addition of carbon dioxide (carbonation of the must) or by sorbic acid. A small quantity of endogenous ethanol is tolerated, with a limit of 1% vol.’ [1].

The preserved musts are widely used to increase the sugar content of low sugar content musts and the addition must be legally permitted. Moreover, they are used to produce concentrated must. As regards the sulfitation, the addition of 100 mg/L of SO_2_ leads to an abrupt stop of fermentation after 24 h [2,3]. Sudraud and Chauvet argued that a molecular SO_2_ content of 1.50 mg/L is necessary to ensure a good stop of fermentation [4].

The addition of SO_2_ in high doses is the simplest way to preserve a must at low energy costs and to kill microorganisms [5]. This type of must is called “mute”. In Italy, one of the best-known wines worldwide, Lambrusco, is produced by using mute must because the high concentration of SO_2_ allows for a greater anthocyanin extraction beyond the must storage for a long period. This must, before being used, needs to be desulfited, and usually this process is performed at 85–95 °C under vacuum [5]. This technique is very expensive from an energy point of view; moreover, the SO_2_ needs to be stripped by using a huge amount of calcium hydroxide, which impacts on the environment and is an expensive process. About 10 years ago, Ferrarini et al. (2009) proposed the use of hydrogen peroxide to desulfite grape must because hydrogen peroxide oxidizes SO_2_ with the production of sulfates by causing an increase in total acidity and ash, lowering wine pH and alkalinity value [6].

Ozone (O_3_) is a potent oxidant compound and although its use for industrial purposes is growing, it is mostly used for the treatment of municipal drinking and wastewater [7]. The solubility of ozone in water is 49.0 mL/100 mL (at 0 °C), ten times higher than oxygen, thus causing an immediate reaction with any biomolecule in biological fluids. In 2011, its use on food was approved by the FDA, whereas in Europe its application is still controversial [8], even though ozone-based food preservation represents a promising green technology for enhanced food safety [9]. It has been used as an oxidant for the reduction and removal of pesticide residues in water [10], in citrus [11], or food in general [12]. Ozone acts as an oxidant in the aqueous oxidation of SO_2_ together with transition metal ion catalysts (${\mathrm{Co}}^{2+}$, ${\mathrm{Fe}}^{3+}$, and ${\mathrm{Mn}}^{2+}$) [13]. SO_2_ can hydrate to produce sulfurous acid (H_2_SO_3_) or ${\mathrm{HSO}}_{3}^{-}$ but the oxidation of H_2_SO_3_ by ozone plays a minor role in the formation of sulfuric acid (${\mathrm{SO}}_{4}^{2-}$) [13]. Also in wine as a hydroalcoholic solution, the addition of SO_2_ produces the bisulfite ion (${\mathrm{HSO}}_{3}^{-}$) and then ${\mathrm{SO}}_{3}^{2-}$ (sulfite form) but the oxidation of the sulfite form provokes the formation of the sulfate ions through the presence of phenol: first, oxygen in the must/wine reacts with a phenolic compound and the oxygen is converted into hydrogen peroxide; then, if free sulfur dioxide is present, the hydrogen peroxide reacts with sulfite to form sulfate [14].

The issue in using hydrogen peroxide is that the more hydrogen peroxide there is, the more sulfate is formed; this is the problem in using H_2_O_2_ for desulfurization.

Ozone has been used in the fuel industry to remove sulfur dioxide [15,16] but to our knowledge no paper has been published on ozone and grape must desulfurization.

Our hypothesis is that the use of ozone permits desulfurization in an environmentally friendly way, without significantly modifying the wine’s pH. On the contrary, a significant color change is expected. To validate our hypothesis, different times of ozone gas exposure of red must were used in measuring the SO_2_ concentration in the must. We also measured the change of the main chemical characteristics and in DO (dissolved oxygen).

## 2. Materials and Methods

### 2.1. Preparation of Tests

To test the effects of ozone, two tests were carried out: the first one involved the use of ozone on a mute Lambrusco must at two temperatures and it was replicated for each temperature. In the second one, deionized/distilled water was used as a medium to which tannins, ascorbic acid and potassium metabisulfite (MBK) were added; this test was replicated several times.

#### 2.1.1. First Test

The mute Lambrusco must was obtained by the addition of sulfur as MBK in high doses to the crushed-destemmed grapes, direct from the Winery Cantine Riunite and CIV, Campegine, Reggio Emilia, Italy. The crushed grapes were then subjected to a sulfite maceration with repeated pumping over at 8-h intervals for 1 or 2 days. At the end of the extraction phase, the crushed grapes were passed through a rough filter which separated the liquid (red mute must) from the solid; the mute must was centrifuged and, after adding other MBK, stored in the tank. The final level of SO_2_ was about 1040 mg/L. The mute must we collected was poured in 4 10 L plastic tanks. The ozone treatment of mute must in this test was carried out at 10 or 20 ± 1 °C in thermocontrolled rooms.

#### 2.1.2. Second Test

The second test was performed at 20 ± 1 °C in two steps. In the first step, 9 4 L glass jars were filled with deionized/distilled water adjusted at pH 3.5 and 50 mg/L of MBK (3 jars), 50 mg/L L-ascorbic acid (Anoxide C, Laffort, Bordeaux, FR) (3 jars), or 1000 mg/L enological tannins (Quertannin line, Laffort, Bordeaux, FR) (3 jars) were added. In the second step, 3 jars of each single compound, MBK, ascorbic acid or tannins, were added with the other two components (mixture) and treated as mentioned earlier. Sampling was performed at time 0 (T0), after 1 h (T1), 3 h (T3), and 5 h (T5). In this test, dissolved oxygen (DO) measurements were performed in glass jars with added MBK, ascorbic acid, or tannins.

### 2.2. Ozone Treatment

The treatment was carried out in both tests with gaseous ozone using an ozone generator from PC Engineering (PC Eng Srl, UggiateTrevano, Italy). O_3_ was dissolved in must or water by the ozone generator which uses ambient air as inlet gas and built-in oxygen concentrator to produce up to max 20 g/h with 6% *w/w* of O_3_ with an ozone concentration of 8 ± 1 µL/L.

In the first test, six 4 L glass jars (three for each temperature), were filled with the red mute must and, through an inlet valve in the sealed cap, ozone gas was bubbled inside at 4 L/min flow rate, allowing the gas to escape through an outlet valve. This flow rate was considered to be the best, based on data by Ratnawati et al. [17]. The outlet tube coming from the ozone generator was split into three jar inlet pipes, guaranteeing for each one a 4 (±0.5) L/min flow rate. The temperature treatment tests were carried out one after the other. The treatment lasted 24 h and must sampling was performed at the start of the ozone treatment (T0) and after 4 h (T1), 7 h (T2) and 24 h (T3). As control, three jars containing mute must were not flushed with ozone but with air.

In the second test, performed only at 20 °C, three glass jars of each sample were treated with ozone gas following the same procedure as before. The test lasted 5 h and sampling was conducted at T0 and after 1, 3, and 5 h.

### 2.3. Analyses

Total sulfur dioxide analyses had been carried out as described by the OIV (Organisation Internationale de la vigne et du vin) in the Compendium [18] and marked with MA-E-AS323-04-DIOSOU. Ascorbic acid was measured by using the colorimetric method by the OIV in the Compendium and marked with MA-E-AS313-13-ALASCO [18]; ascorbic acid is oxidized by iodine to dehydroascorbic acid which is then precipitated using 2,4-dinitrophenylhydrazine to produce bis-(2,4-dinitrophenylhydrazone). After separation by thin layer chromatography and dissolution in an acetic acid medium, the red colored compound is determined by spectrophotometry at 500 nm.

Total polyphenol analysis was carried out by the Folin Ciocalteu method, expressing data as concentration of gallic acid (mg/L).

Color intensity (CI) and tonality (T) had been measured by reading OD (optical density) at 420, 520 and 620 nm as described in Ribereau-Gayon et al. [2,3]: CI = OD_420_ + OD_520_ + OD_620_, T = D420/D520.

The DO (dissolved oxygen) in the glass jar, which was filled completely and tightly sealed with a cap adapted with the oxygen sensor immersed in the deionized/distilled water and the single additives (MBK, ascorbic acid or tannins), was measured by using the oxygen sensor Oxylevel 2200 (Parsec srl, Florence, Italy).

ANOVA was performed for the study of data variance, and the statistical significance of any differences was calculated by the Tukey b test at *p* < 0.01 or 0.05. Calculations were performed in Minitab 15.1, (Minitab, Inc., Coventry, UK).

## 3. Results and Discussion

### 3.1. First Test

**Figure 1:**
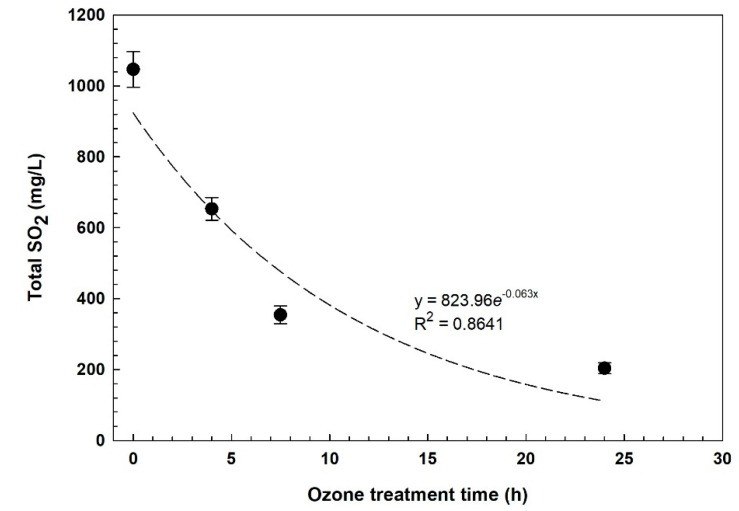
Regression curve of total SO_2_ at 20 °C in the red sulfited must over the ozone treatment time.

Ozone gas bubbled in the mute must significantly reduced the concentration of total SO_2_ (Table 1), and the quadratic regression line with R^2^ indicates the strong chemical reaction between ozone and SO_2_ (Figure 1). In the control jars, no change of SO_2_ was measured at both temperatures.

From the regression curve (Figure 1), it appears that at a high concentration of SO_2_, the effect of ozone is stronger and if we do not consider the last sampling (24 h), a straight line can be drawn and R^2^ is higher than 0.9. After 24 h of treatment, almost 80% of SO_2_ disappeared, by oxidation and evaporation due to bubbling, showing similar results to those obtained by using hydrogen peroxide [5], but, in that case, the test started from a lower concentration (450 mg/L SO_2_) and with a longer treatment time (48 h). Comparing the treatment at two temperatures, as expected, the ozone was more effective at a lower temperature, due to its higher solubility and persistence (Table 1). Free SO_2_ after 24 h was 20 mg/L and 16 mg/L, at 20 and 10 °C, respectively.

Concerning pH (data not shown), an increase from 2.82 to 2.92 after 4 h of treatment was measured. Successively, the value rose to 2.98 at the end of the treatment, regardless of the treatment temperature. The removal of SO_2_ gas could increase the pH as the formation of H_2_SO_3_ following the reaction. SO_2_ in hydroalcoholic solution will consume H^+^ and then the pH would increase. It has been reported that in the presence of O_3_, SO_2_ can be oxidized to sulfate, and this reaction is a first order in SO_2_ and a zero order in O_3_ [19]. It has also been observed, at atmospheric level, that the oxidation of sodium sulfite to sulfate by ozone is faster at higher pH whereas the oxidation with hydrogen peroxide is faster at lower pH [20]. In any case, several factors such as catalysis by metal ions and organic molecules affect the reaction [21].

The effect of ozone on color intensity and tonality is shown in Table 2.

As expected, the strong ozone oxidation provoked a significant change in color from red to orange with increases in CI and T, irrespective of the treatment temperature. This response was confirmed by the significant reduction in total polyphenols (Table 3). No color change and no polyphenol change were observed in the control samples flushed with air for 24 h.

### 3.2. Second Test

To check the potential protection of some antioxidants on the polyphenol decrease, the effect of ozone treatment on MBK, ascorbic acid, and tannins, was tested as a single compound in water solution at wine pH as well as in mixture (Table 4).

The effect of ozone was very strong on sulfur dioxide, causing a decrease of 100% after just 3 h of ozone treatment. The decrease in sulfur dioxide when MBK was used in mixture with tannins and ascorbic acid was an unexpected result; a decrease of 67% was measured after 1 h of ozone treatment. The effect of ozone on tannins was a slight reduction, as single as mixture with ascorbic acid and MBK, after 1 h of ozone treatment, whereas the decrease became significant after 5 h, overall, for the single sample where the decrease was 22% versus only 5% for the mixture. In the case of ascorbic acid as single, the reduction was significant (71%) after just 1 h, reaching a decrease of about 87% after 5 h; when used in mixture with tannins and MBK, the decrease was less than as single after 1 h (53%); after 5 h the decrease was significantly higher (95%).

The additional effect of the other two compounds to MBK appears to increase the oxidation impact of ozone. This is also true for ascorbic acid but only at longer treatment times. Only tannins behaved similarly as single as mixture with a significant oxidation resistance to ozone when ascorbic acid and MBK were added, after just 3 h of ozone treatment.

**Figure 2:**
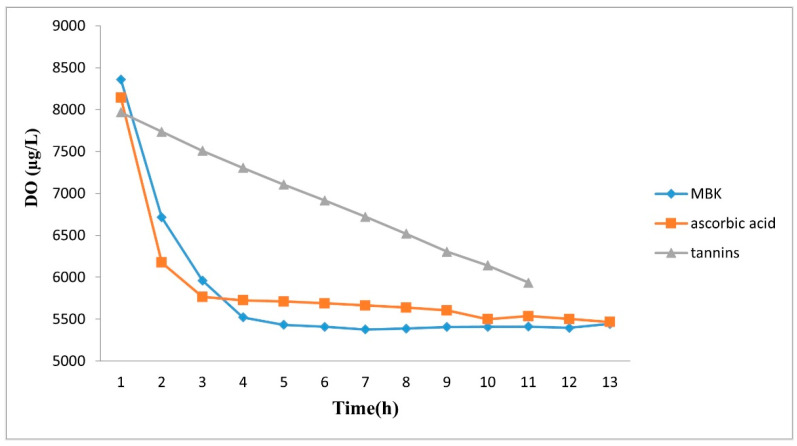
DO patterns in MBK, ascorbic acid and tannins water solutions over the ozone treatment time.

In a preliminary experiment regarding the DO (dissolved oxygen) in deionized water in a closed system, its decrease, when ascorbic acid and MBK were added, was rapid and almost 40% of oxygen was consumed after 3–4 h (Figure 2).

For tannins, the oxygen consumption was a decreasing straight line and, after 11 h, the same values of the DO of the other two compounds were reached. The oxygen formed in water by ozone treatment when it meets organic matter, and especially polyphenols with aromatic ring, decomposes equally in oxygen and OH radicals [22]. Thus, the effect of ozone is due to the high concentration of oxygen but also to the oxygen radical activity. Under oxidative conditions, SO_2_ is consumed to a large extent, with the consequent decrease in its protective action [23]. We do not know if the effect is due to ascorbate or tannins, but it is known that ascorbate can work as pro-oxidant [24,25] and tannins could have a pro-oxidant effect, too. Danilewicz and Wallbridge (2010), argued that sulfite may trap the quinone generated by (+)-catechin oxidation, in this way displacing the redox reaction toward enhanced oxidation and faster consumption of oxygen [26].

Chinnici et al. (2013) showed that the addition of ascorbic acid to a wine model solution containing sulfur dioxide and tannins promptly enhanced the oxygen consumption due to the fast oxidation of ascorbic acid to dehydroascorbic acid and hydrogen peroxide and, as a result, a drastic reduction in the SO_2_ amount [27]. They concluded that ascorbic acid decreased the efficacy of sulfite (by accelerating its oxidation), demonstrating that the former could not be used with the aim to reduce the SO_2_ usage during winemaking. Similar to SO_2_, ascorbate was more oxidized after longer treatment time when MBK and tannins were added. Ascorbic acid is an additive used to prevent oxidation in white wines, due to its fast reaction with molecular oxygen, undergoing preferential oxidation over phenolic compounds [28]. Barril et al. (2012) found a lower consumption of ascorbate in wine-like solutions containing SO_2_, the latter scavenging hydrogen peroxide before this compound could oxidize ascorbate itself [29]. In our experiment this is valid after 1 h of ozone treatment but successively the process of ascorbate loss is accelerated by the presence of MBK and tannins.

In conclusion, although tannins are well protected from the ozone oxidation by the addition of ascorbate and MBK, SO_2_ and ascorbate are not protected by the other two compounds; on the contrary they are pro-oxidized.

## 4. Conclusions

Ozone desulfurization is very efficient in red mute must especially at 10 °C but, as expected, a significant decrease in polyphenols and a significant color change occur. We have tested the effect of ozonation on a water solution with added antioxidants (SO_2_, ascorbic acid, tannins) as single compound or as mixture of the other two; desulfurization was faster with the addition of ascorbate and tannins. Thus, we suggest using ozone desulfurization for white mute must by acting at 10 °C, because the time for the SO_2_ reduction, compared with the traditional physical treatment (high temperature and vacuum), is much shorter, the equipment is less complex (only an ozone generator and a bubbling system), and the energy consumption is significantly reduced (ozone generator consumes less than 1 KW, 220 V), making this technique environmentally friendly and relatively low cost. In addition, no residues are released compared with the use of hydrogen peroxide.

## Figures and Tables

**Table 1:** Content of total SO_2_ in red mute Lambrusco must treated with O_3_ gas (8 µL/L) for the indicated treatment time, at 20 and 10 °C. Data are the mean of three glass jars, each temperature (±SE) in two replicated experiments. In the column, different capital letters indicate significance at *p* < 0.01.

	O_3_ Treatment	Total SO_2_ (mg/L)	
	(h)	T = 20 °C	T = 10 °C
**T0**	0	1040 ± 80 ^A^	1100 ± 90 ^A^
**T1**	4	640 ± 42 ^B^	530 ± 65 ^B^
**T2**	7.5	352 ± 25 ^C^	270 ± 39 ^C^
**T3**	24	200 ± 18 ^D^	120 ± 20 ^D^

**Table 2:** Color intensity (CI) and tonality (T) of red mute Lambrusco must treated with O_3_ gas (8 µL L^−1^) for the indicated time of treatment, at 20 and 10 °C. Data are the mean of three glass jars, each temperature (±SE) in two replicated experiments. In the column for each parameter, different capital letters indicate significance at *p* < 0.01.

	O_3_ Treatment	CI		T	
	(h)	T = 20 °C	T = 10 °C	T = 20 °C	T = 10 °C
**T0**	0	0.773 ± 0.022 ^D^	0.775 ± 0.012 ^D^	366 ± 0.045 ^D^	1.360 ± 0.045 ^D^
**T1**	4	1.187 ± 0.044 ^C^	1.206 ± 0.034 ^C^	2.214 ± 0.066 ^B^	2.010 ± 0.066 ^C^
**T2**	7.5	1.357 ± 0.068 ^B^	1.398 ± 0.048 ^B^	2.180 ± 0.034 ^B^	2.080 ± 0.034 ^C^
**T3**	24	1.522 ± 0.054 ^A^	1.580 ± 0.064 ^A^	3.118 ± 0.038 ^A^	3.198 ± 0.038 ^A^

**Table 3:** Total polyphenols of red mute Lambrusco must treated with O_3_ gas (8 µL/L) for the indicated time of treatment, at 20 and 10 °C. Data are the mean of three glass jars, each temperature (±SE) in two replicated experiments. In the column, different capital letters indicate significance at *p* < 0.01.

	O_3_ Treatment	Total Polyphenols	
	(h)	T = 20 °C	T = 10 °C
**T0**	0	3350 ± 65 ^A^	3430 ± 28 ^A^
**T1**	4	3005 ± 46 ^B^	2970 ± 35 ^B^
**T2**	7.5	2740 ± 45 ^C^	2670 ± 44 ^C^
**T3**	24	2360 ± 39 ^D^	2280 ± 36 ^D^

**Table 4:** Effect of the ozone bubbling (8 µL/L) on the concentration of the indicated compounds as single product or as mixture with the other two compounds. Data are the mean of three glass jars for each sample (±SE), carried out in three to four experiments. In both columns of the indicated compound (SO_2_, tannins, ascorbic acid) as single or mixture, different letters indicate significance at *p* < 0.01 (capital letters) or *p* < 0.05 (lower case letters).

	O_3_ Treatment (h)	SO_2_ (mg/L)	SO_2_ (mg/L) + Mixture	Tannins (mg/L)	Tannins (mg/L) + Mixture	Ascorbic Acid (mg/L)	Ascorbic Acid (mg/L) + Mixture
**T0**	0	28.4 ± 0.6 ^A^	21.9 ± 0.6 ^B^	902 ± 18 ^a^	907 ± 16 ^a^	47.4 ± 2.3 ^A^	50.1 ± 5.5 ^A^
**T1**	1	22.4 ± 0.4 ^B^	7.2 ± 0.2 ^C^	877 ±15 ^abc^	881 ± 16 ^ab^	13.6 ± 1.8 ^C^	27.2 ± 3.2 ^B^
**T2**	3	0	0	746 ± 15 ^d^	864 ± 12 ^bc^	9.3 ± 0.5 ^D^	5.1 ± 0.8 ^E^
**T3**	5	0	0	713 ± 10 ^e^	851 ± 11 ^c^	6.3 ± 1.1 ^E^	2.7 ± 0.6 ^F^

## Data Availability

The data presented in this study are available on request from the corresponding author.

## References

[1] OIV—International Code of Oenological Practices. https://www.oiv.int/en/technical-standards-and-documents/oenological-practices/international-code-of-oenological-practices.

[2] Ribereau-Gayon P., Dubourdieu D., Donèche B., Lonvaud A. (2000). Handbook of Enology: The Microbiology of Wine and Vinifications.

[3] Ribéreau-Gayon P., Glories Y., Maujean A., Dubourdieu D. (2006). Handbook of Enology, The Chemistry of Wine: Stabilization and Treatments.

[4] Sudraud P., Chauvet S., Cazabeil J.-M., Bart M., Crebassa B., Rognon G. (1985). Activité antilevure de l’anhydride sulfureux moléculaire. J. Int. Sci. Vigne Vin.

[5] Catena M. (2014). Aspetti enologici della trasformazione delle uve lambrusco. L’Enologo.

[6] Ferrarini R., Piubelli G., Bocca E., Casarotti E. Studio e messa a punto della tecnica di desolforazione chimica con acqua ossigenata nella produzione di vini elaborati mediante macerazione solfitica. Proceedings of the 32th World Congress of Vine and Wine OIV.

[7] Loeb B.L. (2011). Ozone: Science and engineering: Thirty-three years and growing. Ozone Sci. Eng..

[8] Mencarelli F., Bellincontro A. (2020). Recent advances in postharvest technology of the wine grape to improve the wine aroma. J. Sci. Food Agric..

[9] Pandiselvam R., Subhashini S., Banuu Priya E.P., Kothakota A., Ramesh S.V., Shahir S. (2019). Ozone based food preservation: A promising green technology for enhanced food safety. Ozone Sci. Eng..

[10] Ikehata K., El-Din M.G. (2005). Aqueous pesticide degradation by ozonation and ozone-based advanced oxidation processes: A review (part I). Ozone Sci. Eng..

[11] Kusvuran E., Yildirim D., Mavruk F., Ceyhan M. (2012). Removal of chloropyrifos ethyl, tetradifon and chlorothalonil pesticide residues from citrus by using ozone. J. Hazard. Mater..

[12] Wang S., Wang J., Wang T., Li C., Wu Z. (2019). Effects of ozone treatment on pesticide residues in food: A review. Int. J. Food Sci. Technol..

[13] Sheng F., Jingjing L., Yu C., Fu-Ming T., Xuemei D., Jing-Yao L. (2018). Theoretical study of the oxidation reactions of sulfurous acid/sulfite with ozone to produce sulfuric acid/sulfate with atmospheric implications. RSC Adv..

[14] Danilewicz J.C., Tunbridge P., Kilmartin P.A. (2019). Wine Reduction Potentials: Are These Measured Values Really Reduction Potentials?. J. Agric. Food Chem..

[15] Mok Y.S., Lee H.J. (2006). Removal of sulfur dioxide and nitrogen oxides by using ozone injection and absorption–reduction technique. Fuel Process. Technol..

[16] Nelo S.K., Leskela K.M., Sohlo J.J.K. (1997). Simultaneous oxidation of nitrogen oxides and sulfur dioxide with ozone and hydrogen peroxide. Chem. Eng. Technol..

[17] Ratnawati R., Kusumaningtyas D.A., Suseno P., Prasetyaningrum A. (2018). Mass transfer coefficient of ozone in a bubble column. In Proceedings of 24th Regional Symposium on Chemical Engineering (RSCE 2017), Semarang, Indonesia, 15–16 November 2017. MATEC Web Conf..

[18] OIV—Compendium of International Methods of Analysis of Wines and Musts (Volume 2). https://www.oiv.int/en/technical-standards-and-documents/methods-of-analysis/compendium-of-international-methods-of-analysis-of-wines-and-musts-2-vol.

[19] Li L., Chen M., Zhang Y.H., Zhu T., Li J.L., Ding J. (2006). Kinetics and mechanism of heterogeneous oxidation of sulfur dioxide by ozone on surface of calcium carbonate. Atmos. Chem. Phys..

[20] Penkett S.A., Jones B.M.R., Brice K.A., Eggleton A.E.J. (2007). The importance of atmospheric ozone and hydrogen peroxide in oxidising sulphur dioxide in cloud and rainwater. Atmos. Environ..

[21] Halstead W.D. (1975). A review of saturated vapour pressures and allied data for the principal corrosion products of iron, chromium, nickel and cobalt in flue gases. Corros. Sci..

[22] Travaini R., Martín-Juárez J., Lorenzo-Hernando A., Bolado-Rodríguez S. (2016). Ozonolysis: An advantageous pretreatment for lignocellulosic biomass revisited. Bioresour. Technol..

[23] Danilewicz J.C. (2016). Reaction of oxygen and sulfite in wine. AJEV.

[24] Peng Z., Duncan B., Pocock K.F., Sefton M.A. (1998). The effect of ascorbic acid on oxidative browning of white wines and model wines. Aust. J. Grape Wine Res..

[25] Bradshaw M.P., Cheynier V., Scollary G.R., Prenzler P.D. (2003). Defining the ascorbic acid crossover from anti-oxidant to pro-oxidant in a model wine matrix containing (+)-catechin. J. Agric. Food Chem..

[26] Danilewicz J.C., Wallbridge P.J. (2010). Further studies on the mechanism of interaction polyphenols, oxygen, and sulfite in wine. Am. J. Enol. Vitic..

[27] Chinnici F., Sonni F., Natali N., Riponi C. (2013). Oxidative evolution of (+)-catechin in model white wine solutions containing sulfur dioxide, ascorbic acid or gallotannins. Food Res. Int..

[28] Bradshaw M.P., Barril C., Clark A.C., Prenzler P.D., Scollary G.R. (2011). Ascorbic acid: A review of its chemistry and reactivity in relation to a wine environment. Crit. Rev. Food Sci. Nutr..

[29] Barril C., Clark A.C., Scollary G.R. (2012). Chemistry of ascorbic acid and sulfur dioxide as an antioxidant system relevant to white wine. Anal. Chim. Acta.

